# Supplementary material for: Evaluating the Performance of Widely Used Phylogenetic Models for Gene Expression Evolution
Source: Genome Biol Evol. 2023 Nov 24;15(12):evad211. doi: 10.1093/gbe/evad211 (PMC10709115; doi:10.1093/gbe/evad211)
Supplement: evad211_Supplementary_Data [file evad211_supplementary_data.pdf]

Supplementary materials

Table S1: Number of genes in each dataset best fit to three models.

	Best fit by BM	Best fit by OU	Best fit by EB
AMALGAM	234	1138	5
CAVEFISH	843	2717	0
CICHLIDS	5675	21042	388
FUNGI	1066	2459	31
HELICONIUS	1567	115	711
KMRR	447	7391	495
MAMMALS	1920	3168	232
SULFIDE	10691	5081	968

Table S2: Percentage of genes in each dataset for which the best-fit model performed poorly ($P < 0.05$) as measured by our five test statistics.

Study	$c.var$	$d.cdf$	$s.asr$	$s.hgt$	$s.var$
AMALGAM	7.2%	4.5%	10.1%	0.6%	4.5%
CAVEFISH	6.5%	3.9%	11.6%	0.2%	8.3%
FUNGI	7.8%	4.0%	4.1%	0.6%	7.4%
HELICONIUS	6.0%	4.8%	3.5%	1.0%	5.1%
KMRR	23.1%	13.3%	15.2%	1.9%	14.0%
MAMMALS	17.9%	7.2%	15.6%	1.7%	3.7%
SULFIDE	20.2%	4.4%	15.2%	6.4%	9.2%

Table S3: The number of genes in the CAVEFISH dataset best fit to each model when different normalization methods (CPM, RPKM, TPM) are used.

	Best fit by BM	Best fit by OU	Best fit by EB
CPM	140	451	0
RPKM	143	448	0
TPM	87	504	0

Table S4: The number of fully adequate genes in the CAVEFISH dataset based on different testing statistics when different normalization methods are used.

	c.var	*d.cdf*	*s.asr*	*s.hgt*	*s.var*
CPM	550	562	545	438	546
RPKM	557	560	541	481	557
TPM	564	571	543	385	543

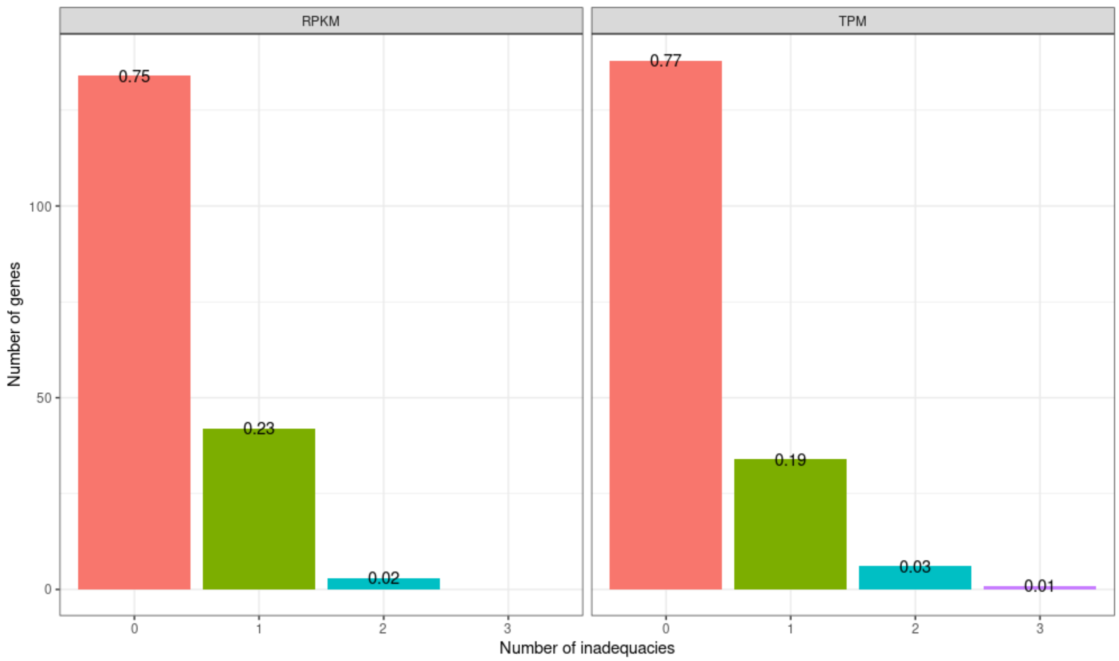

Figure S1: Proportion of inadequate genes for RPKM (left) and TPM (right) normalized reads. Raw reads from the CAVEFISH data set were normalized into RPKM and TPM values and then the best fit model was analyzed for model adequacy via ARBUTUS. The proportion of genes with zero, one, or two inadequacies was nearly identical between both modes of normalization.

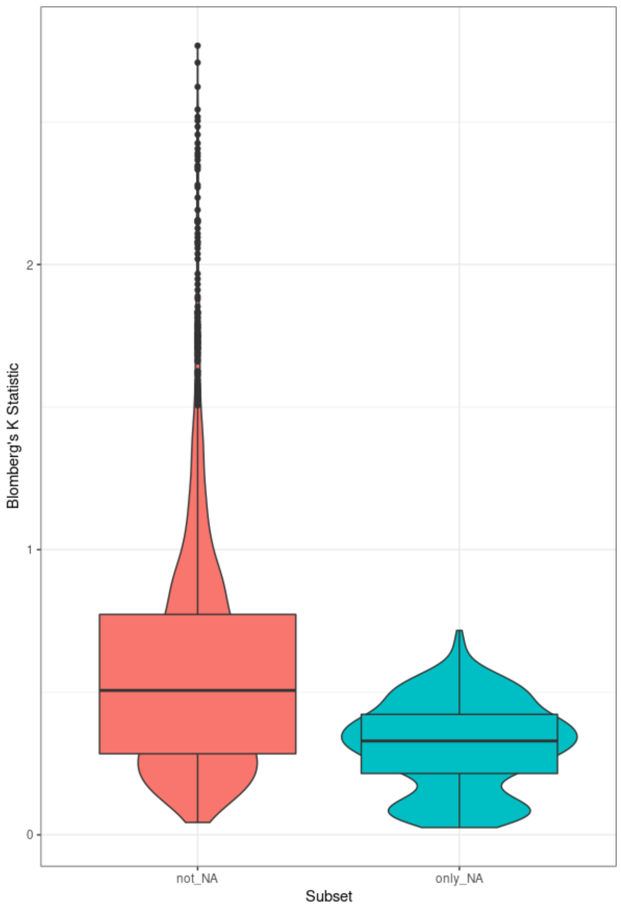

Figure S2: Blomberg's K statistic for genes with NA values (left) and non-NA values (right) in the S.hgt test statistic. Genes with NA values have a lower K statistic on average than genes without.

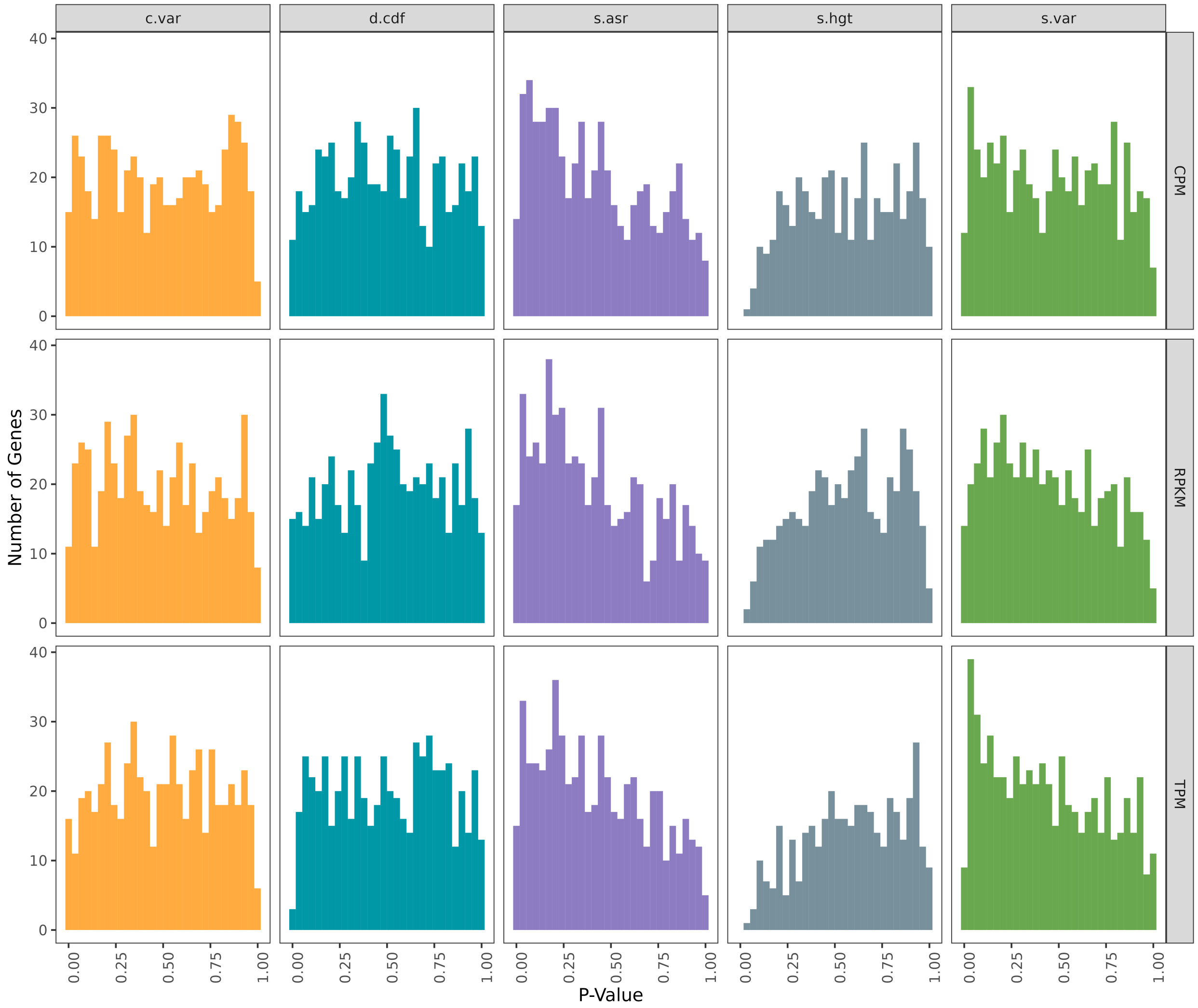

Figure S3: Absolute fit of evolutionary models to the CAVEFISH data set with different normalization methods used. The absolute fit was only evaluated on the (relative) best fit model for each gene. Vertical black lines represent the significance cutoff of 0.05, with an expectation of 5% of genes being inadequate by chance.
